# Supplementary material for: The scientific chaos phase of the great pandemic: A longitudinal analysis and systematic review of the first surge of clinical research concerning COVID-19
Source: PLoS One. 2023 Nov 30;18(11):e0289193. doi: 10.1371/journal.pone.0289193 (PMC10688862; doi:10.1371/journal.pone.0289193)
Supplement: S2 File — (PDF) [file pone.0289193.s002.pdf]

22 items from the original data set downloaded from the WHO ICTRP on the 22nd of April 2020 at 12:57:03 CET.

1. Trial ID
2. Public title
3. Scientific title
4. Primary Sponsor
5. Date of Registration
6. Source Register
7. Recruitment Status
8. Inclusion age minimum
9. Inclusion age maximum
10. Inclusion Gender
11. Date of first enrolment
12. Target Size
13. Study Type
14. Study Design
15. Phase
16. Countries
17. Contact information
18. Inclusion criteria
19. Exclusion criteria
20. (Studied) Health condition
21. Intervention
22. Primary Outcome
