# Supplementary material for: The scientific chaos phase of the great pandemic: A longitudinal analysis and systematic review of the first surge of clinical research concerning COVID-19
Source: PLoS One. 2023 Nov 30;18(11):e0289193. doi: 10.1371/journal.pone.0289193 (PMC10688862; doi:10.1371/journal.pone.0289193)
Supplement: S3 File — (PDF) [file pone.0289193.s003.pdf]

| Item Nr. | Item explanation | Missing or displayed insufficiently if |
|---|---|---|
| 1 | Primary Registry and Trial Identifying Number | No information in original record or dataset from WHO |
| 2 | Date of Registration in Primary Registry | No information in original record or dataset from WHO |
| 3 | Secondary Identifying Numbers | No information in original record or dataset from WHO |
| 4 | Source(s) of Monetary or Material Support | No information in original record or dataset from WHO |
| 5 | Primary Sponsor | No information in original record or dataset from WHO |
| 6 | Secondary Sponsor(s) | No information in original record or dataset from WHO |
| 7 | Contact for Public Queries | "Missing" if no telephone number OR e-mail address available in the dataset and registration record, "not shown on website" if only missing on registration record but available in the dataset from WHO |
| 8 | Contact for Scientific Queries | "Missing" if no telephone number OR e-mail address available in the dataset and registration record, "not shown on website" if only missing on registration record but available in the dataset from WHO |
| 9 | Public Title | No information in original record or dataset from WHO |
| 10 | Scientific Title | No information in original record or dataset from WHO |
| 11 | Countries of Recruitment | No information in original record or dataset from WHO |
| 12 | Health Condition(s) or Problem(s) Studied | No information in original record or dataset from WHO |
| 13 | Intervention(s) | Considered as insufficiently displayed if no generic name, dosage, frequency or duration of the treatment was mentioned OR if a whole arm or control group was only described as standard of care without further information<br><br>Coded as 3 different options: "Intervention name described insufficiently", "Dosage, Frequency or Duration missing" or "SOC as comparator" |
| 14 | Key Inclusion and Exclusion Criteria | No information in original record or dataset from WHO |
| 15 | Study Type | "Missing" if any information on the study design such as method of allocation, masking or assignment was not displayed |
| 16 | Date of First Enrollment | No information in original record or dataset from WHO |

| | | |
|---|---|---|
| 17 | Sample Size | No information in original record or dataset from WHO |
| 18 | Recruitment Status | No information in original record or dataset from WHO |
| 19 | Primary Outcome(s) | No information in original record or dataset from WHO |
| 20 | Key Secondary Outcomes | No information in original record or dataset from WHO |
| 21 | Ethics Review | No information in original record or dataset from WHO |
| 22 | Completion date | No information in original record or dataset from WHO |
| 23 | Summary Results | All trials which already published results were screened to see if they provided summarised results or a link to the publication on their original record |
| 24 | IPD sharing statement | No information in original record or dataset from WHO |
