# Supplementary material for: The scientific chaos phase of the great pandemic: A longitudinal analysis and systematic review of the first surge of clinical research concerning COVID-19
Source: PLoS One. 2023 Nov 30;18(11):e0289193. doi: 10.1371/journal.pone.0289193 (PMC10688862; doi:10.1371/journal.pone.0289193)
Supplement: Attachment — Submitted filename: Response to Reviewers.docx [file pone.0289193.s010.docx]

Response to reviewers

We thank the editorial office and the reviewers for the thorough review of our manuscript and for their thoughtful comments that were very helpful to further strengthening our paper.

In the following section we are addressing each comment point by point:

Journal requirements

Comment 1:

Thank you for stating the following in the Competing Interests section:

“Markus Ries is a guest editor of the call for papers “Prediction and Mitigation of Natural Hazards”.”

Please confirm that this does not alter your adherence to all PLOS ONE policies on sharing data and materials, by including the following statement: "This does not alter our adherence to PLOS ONE policies on sharing data and materials.” (as detailed online in our guide for authors http://journals.plos.org/plosone/s/competing-interests). If there are restrictions on sharing of data and/or materials, please state these. Please note that we cannot proceed with consideration of your article until this information has been declared.

Please include your updated Competing Interests statement in your cover letter; we will change the online submission form on your behalf.

Answer:

We included the updated competing interest statement in the cover letter.

Reviewer #1

Comment 1:

Dear Authors, this is an interesting manuscript, but the Tables 1-6 are cutoff and thus not accessible/readable in the PDF file that I have received for review. Please reformat these items for further review.

Answer:

In our initial submission we strictly complied with the submission guidelines, which state that tables should not be resized and shaped to fit the margins of the manuscript page. Tables that run of those margins can be seen using the “Draft View” of Microsoft Word. Unfortunately, it seems the reviewers have received an automatically created PDF version instead of the Microsoft Word file of the manuscript. We have immediately contacted the PLOS ONE staff team directly, asking for a technical resolution, but unfortunately have not received any answer or solution for this issue so far. To quickly facilitate your important review, we have adjusted all tables to fit the margins of the manuscript page. However, the tables now look very squashed and not as we would like to see in a published version.

Reviewer #2

Comment 1:

This manuscript has great potential to provide some insight into research patterns during the COVID-19 pandemic. The authors explained their methods in great detail, demonstrating the great care they took in identifying both intervention and observational studies.

The authors demonstrated some very important research trends in this study and I think that the comparison to research in other pandemics and high-risk infectious diseases (eg, HIV) was important. I think the authors provide some sound advice to researchers and their research suggests that there has been a great deal of "spinning wheels" as well as some poor quality research churned out, so I like that this paper takes the perspective that we need some caution with COVID-19 research.

Answer:

We would like to express our gratitude for this comment and are extremely pleased for this encouraging statement. We could not agree more.

Comment 2:

A few thoughts for improvement - one thing that was difficult for me was the description of the methods. The methods were very detailed, but a bit long. Are there ways to streamline this? Can the search terms be put into a table, perhaps? That would help the readability of the manuscript and make it easier to follow. I found myself re-reading the methods again and again to be sure I understood what was done. So a bit of streamline could be helpful here.

Answer:

Thank you for this important comment. We totally agree with the reviewer and changed materials and methods to streamline this part. We changed the column of table 1 from “instruments” to “Data sources” and added a new column “variables”. All important information about which data was used on which objective can now be seen in one table. We also added an additional supporting file (S2) with the 22 items which were downloaded from the WHO in the original data set to prevent unnecessary listing of items. We feel that maintaining the reproducibility is very important, which is why we refrained from further streamlining of the methods.

Comment 3:

I had tremendous difficulty with the tables, and honestly, it was a barrier to my providing my review. It was so hard to provide feedback when I couldn't read the full tables. Details are here:

In the uploaded manuscript, table 1 was cut off after DEU column and I was not able to fully assess this table.

In the uploaded manuscript, table 2 was cut off after the "other" column, so I was not able to fully assess this table.

In the uploaded manuscript, table 3 as cutt off after the "other" column, so I was not fully able to assess this table.

In the uploaded manuscript, table 4 was cut off after the EU column, so I was not able to fully assess this table.

In the uploaded manuscript, table 5 was cut off after the EU-CTR column, so I was not fully able to assess this table.

In the uploaded manuscript, table 6 was cut off after the "sequential" column; the column after that starts with the letter N but I could not see any more of this table and therefore I was not able to fully assess it.

I think the issue is that once the Word (or whatever you used to write the manuscript) was converted to a PDF, the formatting of the tables was thrown off and everything was cut off. So that was very hard for me to give feedback on. I am uploading the PDF so you can see how they are cut off.

Answer:

Please see our answer to Reviewer #1, comment 1. We altered the tables and they should now appear fully within the margins of the page. In addition, we are in contact with the PLOS ONE staff to identify the best possible technical solution for this technical conversion issue.

Comment 4:

For the graphs by country, is there any way to have one giant graph of all of the countries together, with different colors representing them? There are just so many tables and a lot to look at. A unified table with the countries together could be instructive and interesting for comparison.

Answer:

Thank you again for this important and helpful critique. We agree with the reviewer and have created one giant graph. Giving each country a different colour would have resulted in a convoluting 20 different colours. As an alternative we decided to work with two colours instead, blue representing the cumulative cases of COVID-19 and orange representing the cumulative trials. As the main message of this graph is to highlight the differences in cases and trial evolution between China and the rest of the world, we highlighted the Chinese graphs and dashed all other graphs. We are convinced that the new graph allows a much better comparison than the previous graphs.
